# A Polygon Model for Wireless Sensor Network Deployment with Directional Sensing Areas

**DOI:** 10.3390/s91209998

**Published:** 2009-12-09

**Authors:** Chun-Hsien Wu, Yeh-Ching Chung

**Affiliations:** Department of Computer Science, National Tsing Hua University, 101, Section 2, Kuang-Fu Road, Hsinchu, 30013, Taiwan; E-Mail: chwu@cs.nthu.edu.tw

**Keywords:** wireless sensor network, sensor node deployment, coverage model, topology control

## Abstract

The modeling of the sensing area of a sensor node is essential for the deployment algorithm of wireless sensor networks (WSNs). In this paper, a polygon model is proposed for the sensor node with directional sensing area. In addition, a WSN deployment algorithm is presented with topology control and scoring mechanisms to maintain network connectivity and improve sensing coverage rate. To evaluate the proposed polygon model and WSN deployment algorithm, a simulation is conducted. The simulation results show that the proposed polygon model outperforms the existed disk model and circular sector model in terms of the maximum sensing coverage rate.

## 1. Introduction

The wireless sensor network (WSN) is a key element of pervasive computing. It provides an efficient way to connect a large number of sensor nodes and collect data from each sensor node. With the advancement of manufacturing technology and wireless communication, many WSN applications have been proposed such as structural health monitoring [1–3], industrial equipment monitoring for petroleum facility [4] and semiconductor plant [5], volcano monitoring networks [6], underwater sensor network [7], habitat monitoring [8], *etc.*

**Figure 1.**
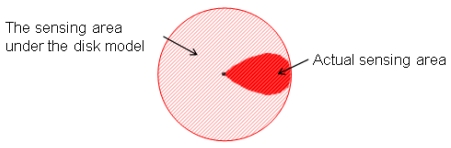
The sensing area of a directional sensor under the disk model.

For all the applications mentioned above, the deployment of sensor nodes is an important issue to deal with. A WSN deployment algorithm has to meet various requirements, such as keeping the network connectivity, maximizing the sensing coverage rate, minimizing the usage of sensor nodes, *etc.* The modeling of the communication and sensing areas of a sensor node is essential to address these requirements. To simplify the complexity, in general, the disk model is used in different aspects of the WSN research [9–11]. However, the round shape coverage area used by the disk model is suitable for the omnidirectional sensor, a sensor with circular sensing area. In this paper, we focus on the directional sensor, a sensor with noncircular sensing area. Figure 1 shows the sensing area of a directional sensor under the disk model. From Figure 1, we can see that the sensing area of a directional sensor is noncircular and at the right side of the sensor. If the disk model is used to represent the noncircular shape, the represented coverage area is larger than the actual one.

**Figure 2.**
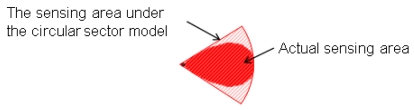
The sensing area of a directional sensor under the circular sector model.

Recently, the circular sector model has been used for representing the sensing area of a directional sensor [12,13]. Figure 2 shows the sensing area of a directional sensor under the circular sector model. From Figure 2, we can see that the actual sensing area cannot be represented by the circular sector model precisely; the sensing area represented by the circular sector model is still larger than the actual one. From the above examples, we can see that the disk model and the circular sector model are not sufficient to model sensor nodes with directional sensors. A new coverage model is needed for sensor nodes with directional sensing areas to improve the accuracy of the WSN deployment results.

In this paper, we propose a polygon model to approximate the sensing area of a directional sensor. The polygon model is composed of a list of vertices expressed in polar coordinates. Since the polygon model can use unlimited number of vertices to model the boundaries of an area, it is more suitable to represent various shapes of sensing areas compared to the disk model and the circular sector model. In addition, a randomized WSN deployment algorithm with topology control mechanism for the polygon model is proposed. The proposed deployment algorithm consists of four steps: the initialization step, the base node selection step, the candidate positions generation step, and the scoring and deployment step. In the initialization step, a deployment area is initialized by reading a configuration file that contains the information of sink nodes and obstacles. In the base node selection step, a base node is selected for deploying new sensor nodes around it. In the candidate positions generation step, some candidate positions for a new sensor node are randomly generated under the topology control mechanism to maintain the network connectivity. In the scoring and deployment step, a new sensor node selected from the deployable sensor nodes is deployed to a candidate position via the scoring process to get the most sensing coverage gains. If no candidate positions are generated in the candidate positions generation step, the deployment algorithm is repeated from the base node selection step to the scoring and deployment step until the sensing coverage rate of the deployment area is 1 or no more sensor nodes can be deployed. Since the sensing coverage rate of the proposed deployment algorithm is determined by the topology control parameter, an iterative approach is used to find the best topology control value that leads to a maximal sensing coverage rate based on the proposed deployment algorithm.

To evaluate the proposed polygon model, some cases are studied based on sensor nodes with different sensing areas (circular or noncircular) and deployment scenarios by using a simulation approach. In the simulation, each sensor node consists of an omnidirectional antenna and a sensor (omnidirectional or directional). The disk model, the circular sector model, and the proposed polygon model are used to model the sensing area of each sensor node and different deployment algorithms are applied to deploy sensor nodes on a field with and without obstacles, respectively. For the sensor nodes represented by the disk model, the algorithm with optimal deployment patterns proposed in [14] is used. For the circular sector model and the polygon model, the proposed WSN deployment algorithm is used. The simulation results show that the proposed polygon model produces much accurate results than the disk model and the circular sector model in terms of the maximum sensing coverage rate in each simulation case. For the proposed WSN deployment algorithm, the maximum sensing coverage rate and the usage of sensor nodes are affected by the values of the topology control parameters and the rotation steps of the sensor node. For the same type of sensor node modeled by a simple polygon (few vertices) and a complex polygon (many vertices), if the number of sensor nodes is sufficient, the difference in maximum sensing coverage rate is very small for sensor nodes modeled by a simple polygon and a complex polygon. However, the usage of sensor nodes under the complex polygon is less than that under the simple polygon.

The rest of the paper is organized as follows. In Section 2, previous works related to circular and noncircular coverage models, sensor node deployment, and topology control are addressed. In Section 3, the polygon model, the calculation of communication/sensing coverage level, and some definitions used in this paper are illustrated. In Section 4, the proposed randomized WSN deployment algorithm and an iterative approach used to find the maximum sensing coverage rate are described in details. Section 5 describes the simulation results of the proposed polygon model under various scenarios.

## 2. Related Work

Each deployment algorithm needs a model to represent the communication and sensing areas of a sensor node. The disk model is the most commonly used model in many research works. It assumes that the communication/sensing area of a sensor node is a circular area. In [9], the authors proposed the *unit disk graph* (UDG) as a coverage model to find a path with maximum observability. In a UDG, there is an edge between two sensor nodes if and only if the Euclidean distance between these two sensor nodes is less than one unit. To provide a more realistic model than the UDG, the *quasi unit disk graph* (Quasi-UDG) was proposes in [10]. In the Quasi-UDG, two sensor nodes are connected by an edge if their distance is less than or equal to a threshold *d*, where *d* is a parameter between 0 and 1. In [15], the authors proposed a model to denote the irregularity of the communication area. The shape of the communication area is controlled by the degree of irregularity (DOI), defined as the maximum radio range variation per unit degree changed from 0° to 360°. When the DOI is set to zero, there is no radio range variation, resulting in a circular communication area the same as the disk model. Based on the DOI model, a radio irregularity model (RIM) based on the empirical data obtained from the MICA2 and MICAZ platforms was proposed in [16]. The RIM provides a general radio model to simulate the degree of radio irregularity. Compare with the DOI model, RIM takes the radio sending energy, the energy loss, the background noise, and the interference among different communication signals into account. Recently, circular sector model is proposed to model the sensing area of a directional sensor node [12,13]. The coverage area under the circular sector model is defined with the central angle and radius, it is identical to the disk model when the central angle is set to 360°. The modeled area under the circular sector model is limited to a sector-like shape while the proposed polygon model in this paper has no such restriction.

Many WSN deployment algorithms and topology control mechanisms based on the disk model have been proposed in the literature. In [17], the authors proposed a regular deployment pattern to achieve both coverage and connectivity. They also extended their work in [14] to provide some optimal regular deployment patterns for full-coverage under different requirements of *k*-connectivity (*k* ≤ 6). A similar regular deployment pattern was proposed in [18], but their approach can deal with the existence of obstacles. The authors in [19] presented a *coverage configuration protocol* (CCP) that can dynamically configure a network to achieve guaranteed degrees of coverage and connectivity. The CCP is based on a simple disk model in which the sensor nodes have identical sensing areas and the communication range between sensor nodes is fixed. In [20], the authors proposed two localized topology control algorithms, *directed relative neighborhood graph* (DRNG) and *directed local spanning subgraph* (DLSS) based on the disk model. In both algorithms, each node independently builds its neighbor set by adjusting the transmission power and defines the network topology by using only local information. Both algorithms preserve network connectivity and network bidirectionality. In [12], the authors proposed the *maximum coverage with minimum sensors* (MCMS) problem that tries to maximize the number of targets to be covered by directional sensor nodes and minimize the number of sensor nodes to be activated. A centralized greedy algorithm (CGA) and a distributed greedy algorithm (DGA) are provided. In [13], the authors proposed two different coverage problems of the directional sensor nodes. One is *connected point-coverage deployment* (CPD), which means covering a set of point-locations with directional sensor nodes. The other is *connected region-coverage deployment* (CRD), which means covering the entire target area with directional sensor nodes. The CRD problem is what we want to deal with in this paper, the authors proposed two regular deployment patterns, *disk-based deployment pattern* (DDP) and *strip-based deployment pattern* (SDP). Both strategies use a similar deployment pattern proposed in [14].

## 3. The Polygon Model

In this paper, we assume that a wireless sensor network consists of one sink node and some sensor nodes of the same type. The sink node contains an omnidirectional antenna (without sensor). Each sensor node contains the same omnidirectional antenna as sink node and a directional sensor. The shapes of the communication areas of the sink node and sensor nodes are circular, and the shapes of the sensing areas of the sensor nodes are noncircular. The definitions of symbols used in this paper are given in Table 1.

In this section, we first describe the polygon model to approximate the shapes of communication and sensing areas of a sensor node. We, then, describe how to calculate the communication and sensing ranges of a sensor node at different directions under the polygon model. Based on the calculated communication and sensing ranges, we can estimate the communication and sensing coverage levels (*CCL* and *SCL*) at any point surrounding a sensor node that can be used by the topology control mechanism of the deployment algorithm. In the following subsections, we will describe them in details.

### 3.1. The Definitions of the Polygon Model

#### Definition 1 (polygon model)

The noncircular shape of the communication/sensing area of a sensor node can be approximated by a list of vertices [(*R*_1_, *θ*_1_), …, (*R_i_, θ_i_*), …, (*R_x_, θ_x_*)], where (*R_i_, θ_i_*) is the *i*th vertex of the list and is represented in polar coordinates, *R_i_* is the radial coordinate and is the distance between the *i*th vertex and the center of a sensor node, *θ_i_* is the angular coordinate and is the counterclockwise angle required to reach the *i*th vertex from 0° (the positive x-axis in the Cartesian coordinate plane), *x* ≥ 3, and 1 ≤ *i* ≤ *x*.

**Figure 3.**
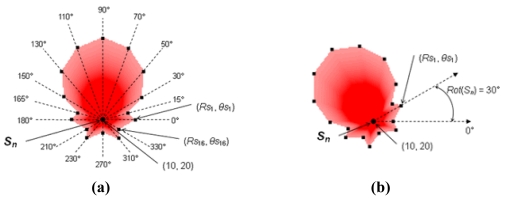
A directional sensing area approximated by the polygon model.

We use *poly_C_*(*S_n_*) = [(*Rc*_1_, *θc*_1_), …, (*Rc_x_, θc_x_*)] and *poly_S_*(*S_n_*) = [(*Rs*_1_, *θs*_1_), …, (*Rs_y_, θs_y_*)] to denote the shapes of communication and sensing areas of a sensor node *S_n_* modeled by the polygon model, respectively. An example of using the polygon model to approximate the noncircular shape of sensing area of a sensor node *S_n_* is given in Figure 3(a). In Figure 3(a), the sensing area of *S_n_* under the polygon model is denoted as *poly_S_*(*S_n_*) = [(*Rs*_1_, *θs*_1_), …, (*Rs*_16_, *θs*_16_)] = [(25, 0°), (20, 15°), (35, 30°), (50, 50°), (60, 70°), (65, 90°), (60, 110°), (50, 130°), (35, 150°), (20, 165°), (25, 180°), (15, 210°), (20, 230°), (10, 270°), (20, 310°), (15, 330°)]. Since the shape of the communication/sensing area of a sensor node *S_n_* is noncircular, when *S_n_* is deployed to a field, the communication/sensing area covered by *S_n_* will depend on the rotation angle of *S_n_*. For example, Figure 3(a) and Figure 3(b) show the sensing areas covered by *S_n_* in a field with rotation angles set to 0° and 30° counterclockwise, respectively. It is obvious that the sensing areas covered by Figure 3(a) and Figure 3(b) are different. We have the following definitions.

#### Definition 2

Given a sensor node *S_n_, Loc*(*S_n_*), *poly_C_*(*S_n_*) and *Rot*(*S_n_*), the communication area covered by *S_n_* in a field under the polygon model is defined as:

(1) $${\text{Area}}_{C}(S_{n})=\{\text{Loc}(S_{n}),[(Rc_{1},+\theta c_{1}+\text{Rot}(S_{n})),\ldots ,(Rc_{x},+\theta c_{x}+\text{Rot}(S_{n}))]\}$$

#### Definition 3

Given a sensor node *S_n_, Loc*(*S_n_*), *poly_S_*(*S_n_*) and *Rot*(*S_n_*), the sensing area covered by *S_n_* in a field under the polygon model is defined as:

(2) $${\text{Area}}_{S}(S_{n})=\{\text{Loc}(S_{n}),[(Rs_{1},+\theta s_{1}+\text{Rot}(S_{n})),\ldots ,(Rs_{y},+\theta s_{y}+\text{Rot}(S_{n}))]\}$$

In Figure 3, if we put sensor node *S_n_* at location (10, 20), then the sensing areas covered by Figure 3(a) and Figure 3(b) in a field are *Area_S_*(*S_n_*) = {(10, 20), [(25, 0°), …,(15, 330°)]} and *Area_S_*(*S_n_*) = {(10, 20), [(25, 30°), …,(15, 360°)]}, respectively.

### 3.2. Calculation of the Communication/Sensing Range

**Figure 4.**
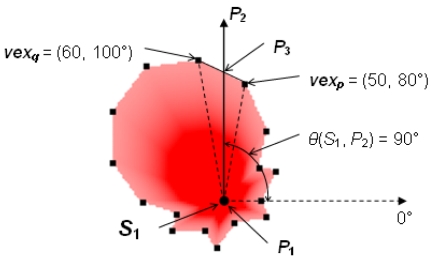
The calculation of *Rs*(*S_n_*, *P_i_*) under the polygon model.

Since the shape of the communication/sensing area of a sensor node is noncircular, the communication/sensing range may be different in different directions. Therefore, we need a formula to calculate the communication and sensing ranges of a sensor node at different directions such that we can determine whether an object is within the communication/sensing area of a sensor node. In Section 3.1, the communication/sensing area is approximated by the polygon model with a list of vertices represented in polar coordinates. Since the radial coordinate of a vertex represents the communication/sensing range of a sensor node in the direction of that vertex, we can use it to compute the communication/sensing range of a sensor node at any direction. Given a point *P_i_*, the sensing area of a sensor node *S_n_, Area_S_*(*S_n_*), and *P_a_* = *Loc*(*S_n_*), if ray *P_a_P_i_* passes between two adjacent vertices of *Area_S_*(*S_n_*), *vex_p_* = (*Rs_p_, θs_p_*) and *vex_q_* = (*Rs_q_, θs_q_*), the sensing range of *S_n_* in the direction of *P_i_* can be calculated by the following equation:

(3) $$R_{S}(S_{n},P_{i})=\frac{\left(Rs_{p}\cdot Rs_{q})\times \sin(\theta s_{q}-\theta s_{p}\right)}{Rs_{p}\times \sin(\theta (S_{n},P_{i})-\theta s_{p})-Rs_{q}\times \sin(\theta (S_{n},P_{i})-\theta s_{q})}$$

where *θs_p_* < *θ*(*S_n_, P_i_*) < *θs_q_, Rs*(*S_n_, P_i_*) = *d*(*P_a_, P_j_*), and *P_j_* is the intersection point of ray *P_a_P_i_* and line segment *vex_p_vex_q_*. The calculation of *Rs*(*S_n_, P_i_*) is based on the area of Δ*vex_p_P_a_vex_q_* that is the sum of the areas of Δ*vex_p_P_a_P_j_* and Δ*P_j_P_a_vex_q_*. The calculation of the communication range of *S_n_* in the direction of *P_i_, Rc*(*S_n_, P_i_*), is similar to Equation (3) by replacing the *Rs* and *θs* parts with *Rc* and *θc*, respectively. Given a sensor node *S*_1_ located at *P*_1_ with *Area_S_*(*S*_1_) shown in Figure 3(b) and a point *P*_2_ with *θ*(*S*_1_, *P*_2_) = 90°, an example of the calculation of *Rs*(*S_n_, P_i_*) is shown in Figure 4. In Figure 4, since ray *P*_1_*P*_2_ passes between *vex_p_* = (50, 80°) and *vex_q_* = (60, 100°) of *Area_S_*(*S*_1_) and *P*_3_ is the intersection point of ray *P*_1_*P*_2_ and line segment *vex_p_vex_q_, Rs*(*S*_1_, *P*_2_) = *d*(*P*_1_, *P*_3_) = (50·60) × sin(100° – 80°)/[50 × sin(90° – 80°) – 60 × sin(90° – 100°)] ≈ 53.7 units.

### 3.3. The Communication/Sensing Coverage Level

**Figure 5.**
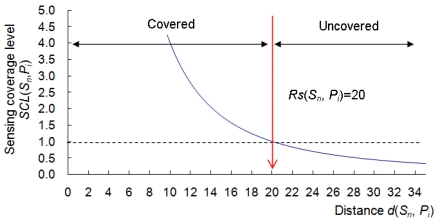
The relationship between *SCL*(*S_n_*, *P_i_*) and *d*(*S_n_*, *P_i_*).

Based on the calculated communication and sensing ranges, we can estimate the communication coverage level (*CCL*) and sensing coverage level (*SCL*) at any point surrounding a sensor node. *CCL* and *SCL* are used by the topology control mechanism in the proposed WSN deployment algorithm and the calculation of the sensing coverage rate. The calculation of the communication/sensing coverage level is based on the free space propagation model proposed in [21], a simple transmission formula for a radio circuit is derived:

(4) $$\frac{{\text{Power}}_{r}}{{\text{Power}}_{t}}=\frac{{\text{Area}}_{r}\cdot {\text{Area}}_{t}}{d^{2}\cdot \lambda^{2}}$$

where *Power_t_* is the power fed into the transmitting antenna at its input terminals, *Power_r_* is the power available at the output terminals of the receiving antenna, *Area_r_* (or *Area_t_*) is the effective area of the receiving (or transmitting) antenna, *d* is the distance between two antennas, and *λ* is the wavelength. Assume that *Power_t_, Area_r_, Area_t_*, and *λ* are constants in Equation (4). The received radio power (*Power_r_*) is proportional to 1/*d*^2^. The *CCL* and *SCL* of a sensor node *S_n_* at a point *P_i_* are defined as:

(5) $$\text{CCL}(S_{n},P_{i})={\left[Rc(S_{n},P_{i})/d(S_{n},P_{i})\right]}^{2}$$

(6) $$\text{SCL}(S_{n},P_{i})={\left[Rs(S_{n},P_{i})/d(S_{n},P_{i})\right]}^{2}$$

where *Rc*(*S_n_, P_i_*) and *Rs*(*S_n_, P_i_*) are the communication and sensing ranges of *S_n_* in the direction of *P_i_*, and *d*(*S_n_, P_i_*) is the Euclidean distance between *S_n_* and *P_i_*. Figure 5 shows the relationship between *SCL*(*S_n_, P_i_*) and *d*(*S_n_, P_i_*). In Figure 5, if *d*(*S_n_, P_i_*) ≤ *Rs*(*S_n_, P_i_*), it indicates that *P_i_* is within the sensing area of *S_n_*. Otherwise, *P_i_* is not covered by the sensing area of *S_n_*. In the case of a deployment area with obstacles, the calculation of the *CCL* and *SCL* follows the “line-of-sight” property, that is, if a straight line between *S_n_* and *P_i_* is blocked by an obstacle, then *CCL*(*S_n_, P_i_*) = *SCL*(*S_n_, P_i_*) = 0.

### 3.4. Network Connectivity and Sensing Coverage Rate

A communication-connected WSN is one of the objectives of deployment. We have the following definitions for network connectivity.

#### Definition 4

Two sensor nodes, *S*_1_ and *S*_2_, located at point *P*_1_ and *P*_2_, respectively, are connected if *CCL*(*S*_1_, *P*_2_) ≥ 1 and *CCL*(*S*_2_, *P*_1_) ≥ 1.

#### Definition 5 (network connectivity)

Given a WSN *W* in a field, the network connectivity of *W, NC*(*W*), is defined as:

(7) $$NC(W)=\text{Num}(S_{n}\;\text{in}\;W\;\text{has a path to the sink node and vice versa})/\text{Num}(S_{n}\;\text{in}\;W)$$

where *Num*(*S_n_* in *W* has a path to the sink node and vice versa) is the number of senor nodes in *W* that has a path to the sink node and vice versa; and *Num*(*S_n_* in *W*) is the number of sensor nodes in *W*.

#### Definition 6

A WSN *W* is communication-connected if *NC*(*W*) = 1.

The sensing coverage rate is a metric to evaluate the performance of a WSN deployment algorithm. The higher sensing coverage rate, in general, the superior of a WSN deployment algorithm. To calculate the sensing coverage rate, we have the following definitions for sensing coverage rate.

#### Definition 7

A grid point *P_i_* in a field is sensed by a sensor node *S_n_* if *SCL*(*S_n_, P_i_*) ≥ 1.

#### Definition 8 (sensing coverage rate of a field)

Given a field, *Area*, the sensing coverage rate of a WSN *W* on *Area* is defined as:

(8) $$CR(\text{Area},W)=\text{Num}(P_{x}\;\text{in}\;\text{Area}\;\text{sensed by sensor nodes in}\;W)/\text{Num}(P_{y}\;\text{in}\;\text{Area})$$

where *Num*(*P_x_* in *Area* sensed by sensor nodes in *W*) is the number of grid points in *Area* sensed by sensor nodes in *W* and *Num*(*P_y_* in *Area*) is the number of grid points in *Area*.

#### Definition 9 (sensing coverage rate of a square area centered at grid point *P_i_*)

Given a grid point *P_i_*, the sensing coverage rate of a WSN *W* on the square area centered at grid point *P_i_, Square*(*P_i_*) is defined as:

(9) $$CR(\text{Square}(P_{i}),W)=\text{Num}(P_{x}\;\text{in}\;\text{Square}(P_{i})\;\text{sensed by sensor nodes in}\;W)/\text{Num}(P_{y}\;\text{in}\;\text{Square}(P_{i}))$$

where *Num*(*P_x_* in *Square*(*P_i_*) sensed by sensor nodes in *W*) is the number of grid points in *Square*(*P_i_*) sensed by sensor nodes in *W* and *Num*(*P_y_* in *Square*(*P_i_*)) is the number of grid points in *Square*(*P_i_*).

## 4. A Randomized WSN Deployment Algorithm for the Polygon Model

The proposed deployment algorithm for the polygon model consists of four steps:

1. **Initialization:** In this step, the deployment parameters, a deployment area with or without obstacles, and a sink node (used for collecting sensing data) are initialized by reading a configuration file;
2. **Base node selection:** In this step, a base node is selected for deploying new sensor nodes around it;
3. **Candidate positions generation:** In this step, some candidate positions for new sensor nodes are generated under the topology control mechanism to maintain the network connectivity;
4. **Scoring and deployment:** In this step, a new sensor node is deployed to a candidate position via the scoring process.

In the above procedure, Step 3 and Step 4 are repeated until no candidate positions can be generated in Step 3. Then the deployment algorithm is restarted from Step 2 until the sensing coverage rate of the deployment area is 1 or no more sensor nodes can be deployed. The detailed deployment steps are described in the following subsections.

### 4.1. Step 1: Initialization

Given a configuration file, some deployment parameters are set, including the number of deployable sensor nodes, the limit of candidate positions, and the number of rotation steps. The deployment area is initialized based on the following information:

1. The length and the width of the deployment area;
2. Information of the sink node, such as the location, the rotation angle, and its communication area approximated by the polygon model;
3. Information of the obstacles (optional), where each obstacle is a polygon represented by a list of the vertices.

**Figure 6.**
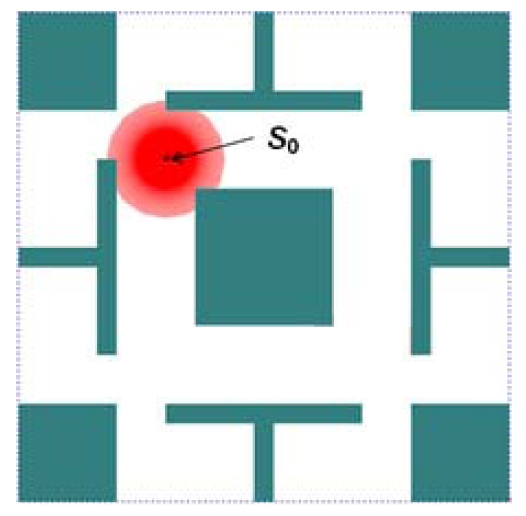
An initialized deployment area.

Figure 6 shows an example of the initialized deployment area. The deployment area contains a sink node (*S*_0_) and the red area represents the communication area of *S*_0_. Some obstacles with different shapes are distributed in the deployment area as well.

### 4.2. Step 2: Base Node Selection

**Figure 7.**
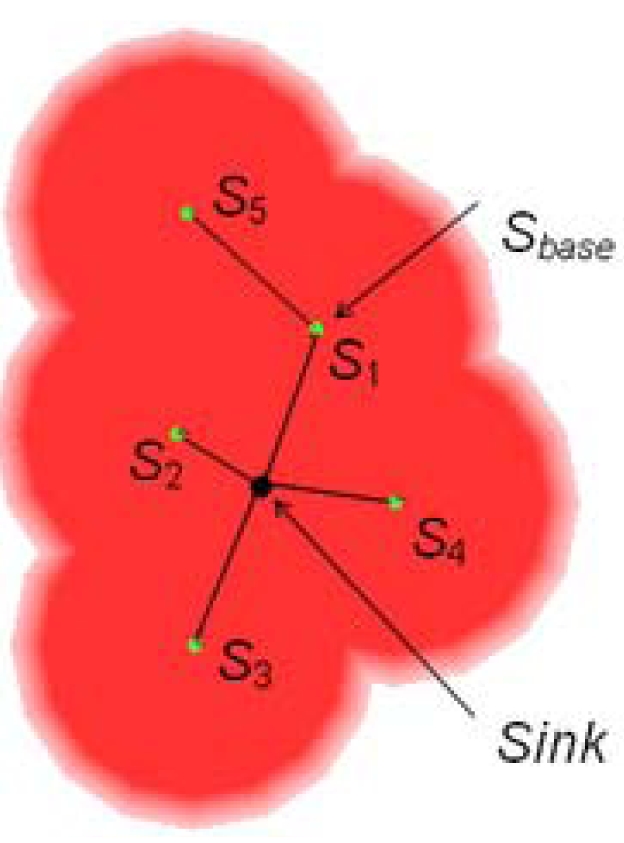
An example of base node selection.

In this step, a base node *S_base_* is selected for deploying new sensor nodes around it. In order to maintain the network connectivity of a WSN, the selection of base node is starting from the sink node and traversing all deployed sensor nodes along the communication links. When no more sensor nodes can be deployed around the current base node, a new base node is selected from deployed sensor nodes based on their index in ascending order. An example of base node selection is given in Figure 7. In Figure 7, four sensor nodes, *S*_1_, *S*_2_, *S*_3_, and *S*_4_, are deployed when the sink node is selected as base node. *S*_1_ is the current base node. When *S*_5_ is deployed, no more sensor nodes can be deployed around *S*_1_. *S*_2_ is then selected as the new base node.

### 4.3. Step 3: Candidate Positions Generation and Topology Control Mechanism

Once a base node *S_base_* is selected, a predefined number of candidate positions for a new sensor node are randomly generated within the communication area of *S_base_* based on the topology control mechanism. The limit of candidate positions is defined in configuration file. If a candidate position cannot pass the examination of the topology control mechanism, another candidate position will be generated. The purpose of the topology control mechanism is to keep the candidate position not too close to the deployed sensor nodes. As a result, the overlap of sensing areas among the deployed sensor nodes can be reduced.

**Figure 8.**
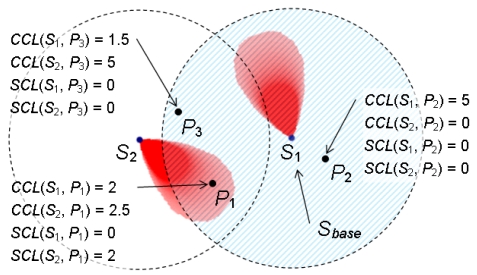
An example of the topology control mechanism.

Given a candidate position *P_i_* within *Area_C_*(*S_base_*) and a set of sensor nodes deployed around *P_i_, S_depolyed_*, the topology control mechanism is performed as follows. For each sensor node *S_n_* in *S_depolyed_*, the communication coverage level of *S_n_* at *P_i_, CCL*(*S_n_, P_i_*), is calculated based on Equation (5). If one of *CCL*(*S_n_, P_i_*) exceeds the predefined topology control parameter Max_CCL, the maximum threshold of the communication coverage level, *P_i_* will be abandoned since *P_i_* is too close to the location of *S_n_*. Next, for *P_i_* with *CCL*(*S_n_, P_i_*) ≤ *Max*_*CCL* for all *S_n_* in *S_depolyed_*, the sensing coverage level of *S_n_* at *P_i_, SCL*(*S_n_, P_i_*), is calculated based on Equation (6). If one of *SCL*(*S_n_, P_i_*) exceeds another predefined topology control parameter *Max*_*SCL*, the maximum threshold of the sensing coverage level, *P_i_* will be abandoned. Figure 8 shows an example of the process of the topology control mechanism. In Figure 8, two deployed sensor nodes *S*_1_ and *S*_2_ are given. *S*_1_ is selected as *S_base_* and three candidate positions *P*_1_, *P*_2_, and *P*_3_ are randomly generated within the communication area of *S_base_* (represented as a circular area centered at *S*_1_). Assume that *Max*_*CCL* = 4 and *Max*_*SCL* = 3. Since *CCL*(*S*_1_, *P*_2_) = *CCL*(*S*_2_, *P*_3_) = 5 > *Max*_*CCL, P*_2_ and *P*_3_ are abandoned. *P*_1_ is kept since *CCL*(*S*_1_, *P*_1_) and *CCL*(*S*_2_, *P*_1_) are less than *Max*_*CCL*, and *SCL*(*S*_2_, *P*_1_) = 2 < *Max*_*SCL*.

The thresholds of the topology control parameters, *Max*_*CCL* and *Max*_*SCL*, will affect the results of deployment. The value of *Max*_*CCL* is never less than 1 in order to maintain the network connectivity between sensor nodes. If *Max*_*CCL* is set too large, the minimal distance between deployed sensor nodes will be too short. More sensor nodes will be needed to fully cover the same deployment area compared to the optimal case. The value of *Max*_*SCL* varies with *Max*_*CCL* if the sensor node has an omnidirectional sensor. Given the value of *Max*_*CCL* and the maximum communication and sensing ranges of a sensor node, *Rc* and *Rs*, the value of *Max*_*SCL* is defined as:

(10) $$\text{Max}\_\text{SCL}={\left[\frac{Rs}{Rc}\right]}^{2}\times \text{Max}\_\text{CCL}$$

If the sensor node has a directional sensor, more sensor nodes are needed to cover the same deployment area compared to the sensor node has an omnidirectional sensor with the same maximum sensing range. Therefore, the value of *Max*_*SCL* is not changed with *Max*_*CCL* since a larger *Max*_*CCL* is needed to make the sensor nodes get closer. We have two cases for the value of *Max*_*SCL*.

#### Case 1 (*Rc* ≥ *Rs*)

In this case, the value of *Max*_*SCL* is set to 1 to reduce the overlap of sensing areas by preventing a new sensor node being deployed within the sensing areas of the deployed sensor nodes.

#### Case 2 (*Rc* < *Rs*)

In this case, in order to maintain the network connectivity, the value of *Max*_*SCL* is set to (*Rs/Rc*)^2^ to allow the overlap of sensing areas between a new sensor node and deployed sensor nodes. For example, as shown in Figure 8, if we set *Max*_*CCL* = 6 and *Max*_*SCL* = 1, *P*_1_ is abandoned since *SCL*(*S*_2_, *P*_1_) = 2 > *Max*_*SCL*. *P*_2_ and *P*_3_ can be selected as new positions of sensor nodes.

### 4.4. Step 4: Scoring and Deployment

In this step, a scoring process is applied to each candidate position of {*P*_1_, …, *P_k_*} generated in Step 3. Given a sensor node *S_new_*, a candidate position *P_i_* in {*P*_1_, …, *P_k_*}, and the WSN *W* constructed so far, the score of *P_i_, Score*(*P_i_*), is defined as:

(11) $$\text{Score}(P_{i})=CR(\text{Square}(P_{i}),W\cup S_{\text{new}})-CR(\text{Square}(P_{i}),W)$$

where *Square*(*P_i_*) is a square area centered at *P_i_* with edge length = 2 × *Rs. CR*(*Square*(*P_i_*), *W* ∪ *S_new_*) and *CR*(*Square*(*P_i_*), *W*) are the sensing coverage rates of *W* on *Square*(*P_i_*) before and after *S_new_* is deployed at *P_i_*, respectively. Since the sensing area of a sensor node is noncircular, to deploy *S_new_* at *P_i_* may result in different values of *Score*(*P_i_*) if *S_new_* at *P_i_* are rotated by different angles counterclockwise from 0°. Therefore, we have two cases for the calculation of *Score*(*P_i_*).

#### Case 1 (without rotation)

In this case, *Score*(*P_i_*) is only computed once using Equation (11).

#### Case 2 (with rotation)

In this case, the number of rotation steps, *Rot_step_*, is specified. Given *Rot_step_* defined in the configuration file, when *S_new_* is deplo yed at *P_i_*, there are *Rot_step_* values of *Score*(*P_i_*) with respect to the rotation degree 0°, (360°/*Rot_step_*), (360°/*Rot_step_*) × 2, …, and (360°/*Rot_step_*) × (*Rot_step_*–1), respectively. If *S_new_* has a noncircular communication area, the rotation of *S_new_* also affects the communication area of *S_new_* at *P_i_*. The network connectivity of WSN needs to be maintained when *S_new_* is rotated an angle counterclockwise from 0°. Given a specific rotation angle *θ*, if the network connectivity of WSN can be maintained after *S_new_* is deployed at *P_i_* by rotating *S_new_* with an angle *θ* counterclockwise from 0°, *Score*(*P_i_*) can be obtained by using Equation (11). Otherwise, *Score*(*P_i_*) = 0. After different *Score*(*P_i_*) for different rotation angles are calculated, the highest score of *Score*(*P_i_*) is kept and the corresponding *Rot*(*S_new_*) is saved.

**Figure 9.**
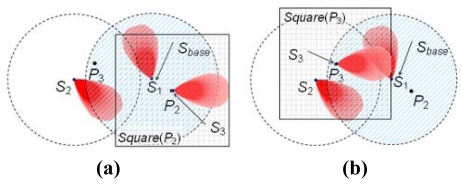
An example of the scoring process for Case 1.

Figure 9 gives an example of the scoring process for Case 1. In Figure 9, a base node *S*_1_ and a set of candidate positions {*P*_2_, *P*_3_} are given. Before deploying a new sensor node *S*_3_ at *P*_2_, *CR*(*Square*(*P*_2_), *W*) = 0.09. After *S*_3_ is deployed at *P*_2_ as shown in Figure 9(a), *CR*(*Square*(*P*_2_), *W* ∪ *S*_3_) = 0.22. We obtain *Score*(*P*_2_) = 0.22 – 0.09 = 0.13. Similarly, before deploying *S*_3_ at *P*_3_, *CR*(*Square*(*P*_3_), *W*) = 0.249. After *S*_3_ is deployed at *P*_3_ as shown in Figure 9(b), *CR*(*Square*(*P*_3_), *W* ∪ *S*_3_) = 0.329. We have *Score*(*P*_3_) = 0.329–0.249 = 0.08. Since *Score*(*P*_2_) > *Score*(*P*_3_), *P*_2_ is the best candidate position for deploying *S*_3_ in this example.

After the scoring process is applied to each candidate positions, the best candidate position *P_i_* is the one with the largest value of *Score*(*P_i_*). It means that the deployment of *S_new_* at *P_i_* can have the most gains of the sensing coverage rate. Then, *S_new_* is deployed at *P_i_* and the deployment flow goes back to Step 3. If no candidate positions can be generated in Step 3 for scoring, the deployment algorithm is restarted from Step 2 (base node selection) until the sensing coverage rate of the deployment area is 1 or no more sensor nodes can be deployed. The details of the proposed deployment algorithm for the polygon model, *Deploy-Random*, is given below:
***Algorithm** Deploy-Random*(*num_deployable, limit_candidate, Max_CCL, sen_covrate, num_deployed*)/*Step 1: Initialization */1.Initialize deployment area;2.Add sink node to *Node_deployed_* and *Node_base_*;3.Calculate *Max_SCL* based on *Max_CCL* and type of sensor;4.**while** (*Node_base_* is not empty){  /* Step 2: Base node selection */5.Select a *S_base_* from *Node_base_*;6.*base_deployable* = true;7.**while** (*base_deployable* is true){   /* Step 3: Candidate positions generation */8.Clear *Candidate* and set *num_candidate* = 0;9.**while** (*num_candidate* < *limit_candidate*){10.Generates a position *P_i_* randomly within *Area_C_*(*S_base_*);11.**if** (*P_i_* is eligible under the topology control mechanism with *Max_CCL* and *Max_SCL*){12.Add *P_i_* to *Candidate*;13.*num_candidate*++;14.}15.}16.**if**(*num_candidate* == 0)*base_deployable* = false;/* select a new *S_base_* */17.else{/* Step 4: Scoring and deployment */18.Generate a new sensor node *S_new_*;19.for (all *P_i_* in *Candidate*)Call *Score*(*P_i_*) and save score to *CandidateScore*[*P_i_*];20.Deploy *S_new_* to *P_i_* that has the highest score in *CandidateScore*;21.Add *S_new_* to *Node_deployed_* and *Node_base_*;22.*num_deployable*--;23.**if** (*num_deployable* == 0)**break**;24.}25.}26.Remove *S_base_* from *Node_base_*;/* restart from Step 2 */27.}28.**return***sen_covrate* and *num_deployed*;*End_of_Deploy-Random*

The time complexity of algorithm *Deploy-Random* is *O*(*N* * (*T_Cand_* + *T_Score_*)), where *N* is the number of deployable sensor nodes, *T_Cand_* = *O*(*Area_C_*(*S_base_*) * *N*) is the time to generate candidate positions stated in Step 3 of the algorithm, and *T_Score_* = *O*(*Rs*^2^) is the time of scoring stated in Step 4 of the algorithm.

### 4.5. Find the Maximum Sensing Coverage Rate

In algorithm *Deploy-Random*, the sensing coverage rate is affected by the topology control parameter, *Max_CCL*. To find the maximum sensing coverage rate, an algorithm *Find-Max-Sen-Covrate* is used by adjusting the value of *Max_CCL* iteratively. The details of algorithm *Find-Max-Sen-Covrate* is given below:
***Algorithm****Find-Max-Sen-Covrate*(*num_deployable, limit_candidate, min_CCL_diff*)/*Initialization */1.*LB_CCL* = (*Rc/d_max_*)^2^;/* *d_max_* is the maximum distance between sensor nodes */2.*UB_CCL* = (*Rc/d_min_*)^2^;/* *d_min_* is the minimum distance between sensor nodes *//*Test with lower bound */3.*Deploy-Random*(*num_deployable, limit_candidate, LB_CCL, LB_sen_covrate, LB_num_deployed*);4.**if** (*LB_sen_covrate* = 1 ‖ *LB_num_deployed* = *num_deployable*) **return***LB_sen_covrate*;/*Test with upper bound */5.*Deploy-Random*(*num_deployable, limit_candidate, UB_CCL, UB_sen_covrate, UB_num_deployed*);6.**if** (*UB_sen_covrate* < 1 && *UB_num_deployed* < *num_deployable*) **return***UB_sen_covrate*;/*Test with medium */7.**if** ((*UB_CCL* − *LB_CCL*) < *min_CCL_diff*) **return***Max*(*UB_sen_covrate, LB_sen_covrate*);8.*MB_CCL* = (*LB_CCL*+*UB_CCL*)/2;9.*Deploy-Random*(*num_deployable, limit_candidate, MB_CCL, MB_sen_covrate, MB_num_deployed*);10.**if** (*MB_sen_covrate* < 1 && *MB_num_deployed* < *num_deployable*)  { *LB_CCL* = *MB_CCL, LB_sen_covrate* = *MB_sen_covrate*,  *LB_num_deployed* = *MB_num_deployed* }11.**else** { *UB_CCL* = *MB_CCL, UB_sen_covrate* = *MB_sen_covrate*,  *UB_num_deployed* = *MB_num_deployed* }12.**Go to** line 7;*End_of_Find-Max-Sen-Covrate*

In algorithm *Find-Max-Sen-Covrate*, initially, the lower bound and the upper bound of *Max_CCL* are set to *LB_CCL* = (*Rc/d_max_*)^2^ and *UB_CCL* (*Rc/d_min_*)^2^ based on Equation (5) in Section 3.3, respectively. By calling algorithm *Deploy-Random* at line 3, we can get the sensing coverage rate, *LB_sen_covrate*, and the number of deployed sensor nodes, *LB_num_deployed*, for *Max_CCL* = *LB_CCL*. If *LB_sen_covrate* = 1 or *LB_num_deployed* = *num_deployable*, it means that either a deployment area is fully covered or all sensor nodes will also be deployed when higher *Max_CCL* values are specified. In this case, *LB_sen_covrate* is the maximum sensing coverage rate. Otherwise, algorithm *Deploy-Random* at line 5 is called to get the sensing coverage rate, *UB_sen_covrate*, and the number of deployed sensor nodes, *UB_num_deployed*, for *Max_CCL* = *UB_CCL*. If *UB_sen_covrate* < 1 and *UB_num_deployed* < *num_deployable*, it indicates that no more sensor nodes can be deployed to the deployment area even though there are some deployable senor nodes available. In this case, *UB_sen_covrate* is the maximum sensing coverage rate. If the maximum sensing coverage rate is not found at the lower and upper bounds of *Max_CCL*, it must in between *LB_CCL* and *UB_CCL*. Therefore, a binary search approach is applied to the range of (*LB_CCL, UB_CCL*) in order to find the value of *Max_CCL* that lead to the maximum sensing coverage rate. Since *LB_CCL* and *UB_CCL* are real numbers, the number of binary search iterations is infinite. Therefore, a minimum distance of the lower and upper bounds, *min_CCL_diff*, is specified. If *UB_CCL* − *LB_CCL* < *min_CCL_diff, Max*(*UB_sen_covrate, LB_sen_covrate*) is the maximum sensing coverage rate. Otherwise, algorithm *Deploy-Random* at line 9 is applied with a new *Max_CCL* = (*LB_CCL*+*UB_CCL*)/2 to get the sensing coverage rate, *MB_sen_covrate*, and the number of deployed sensor nodes, *MB_num_deployed*. If *MB_sen_covrate* < 1 and *MB_num_deployed* < *num_deployable*, it indicates that there exists a value in between (*LB_CCL*+*UB_CCL*)/2 and *UB_CCL* whose sensing coverage rate is greater than *MB_sen_covrate*. Then lines 7–12 are repeated for the range ((*LB_CCL*+*UB_CCL*)/2, *UB_CCL*). Otherwise, there exists a value in between *LB_CCL* and (*LB_CCL*+*UB_CCL*)/2 whose sensing coverage rate is greater than *MB_sen_covrate*. Lines 7–12 are repeated for the range (*LB_CCL*, (*LB_CCL*+*UB_CCL*)/2). The time complexity of the algorithm *Find-Max-Sen-Covrate* is *O*(*log*_2_(*Rc*^2^) * *T_Deploy_*), where *T_Deploy_* is the time complexity of algorithm *Deploy-Random*.

## 5. Deployment Simulation

To evaluate the proposed polygon model and *Deploy-Random* algorithm, the deployment simulation is divided into three parts. In the first part, we compare the deployment results when a sensor node is modeled with the disk model, the circular sector model, and the proposed polygon model, respectively. In the second part, we compare the difference of the deployment results when a sensor node is modeled by the polygon model and the *Deploy-Random* algorithm is applied with different rotation steps. In the third part, we compare the difference of the deployment results when a sensor node is modeled by the polygon model with different number of vertices.

### 5.1. Simulation 1: Compare with the Disk Model and the Circular Sector Model

In Simulation 1, there are two types of sensor nodes used. They are defined as follows:

Type 1: omnidirectional antenna + omnidirectional sensor, and
Type 2: omnidirectional antenna + directional sensor.

**Figure 10.**
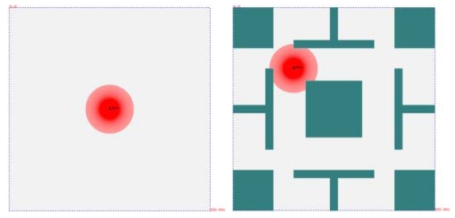
Two deployment areas used in the simulation.

Table 2 and 3 shows the shapes of the communication/sensing areas of omnidirectional antenna/sensor and directional sensor, and the detailed modeling information for each type of sensor node under the disk model, circular sector model, and the polygon model. From Table 2 and 3, we can see that the communication and sensing areas of the omnidirectional antenna and the omnidirectional sensor are round shapes. The sensing area of the directional sensor is noncircular shape. Therefore, sensor nodes of Type 1 are generally modeled with disk model. Sensor nodes of Types 2 have noncircular sensing areas are suitable modeled with the circular sector model or the proposed polygon model. To get a fair comparison, a disk model based deployment algorithm is used by the sensor nodes under the disk model. The *Deploy-Random* algorithm is used by the sensor nodes under the circular sector model and the polygon model. The number of sensor nodes for each type is set to 1000 and the limit of candidate positions is set to 3. For the deployment area, one with obstacles and the other without obstacles, as shown in Figure 10, are used. The size of both deployment areas is 500 × 500 units. A sink node (*S*_0_) has an omnidirectional antenna with communication range 60 is deployed before simulation. In the deployment area without obstacles, *S*_0_ is deployed at (250, 250). In the deployment area with 9 obstacles, *S*_0_ is located at (150, 150).

To get the deployment results, each type of sensor nodes are deployed to the deployment areas using the disk model based algorithm proposed in [14] with 6-connectivity, and the proposed *Deploy-Random* algorithm. For the disk model based deployment algorithm, the deployment pattern of sensor nodes is decided by the ratio of the maximum communication range and the maximum sensing range (*Rc/Rs*). Therefore, three different *Rc/Rs* ratios, 2 (60/30), 1.2 (60/50), and 1/1.2 (60/72), are simulated for each type of sensor node base on [14]. For the *Deploy-Random* algorithm, the same *Rc/Rs* ratios as those of the disk model based deployment algorithm are used and with the rotation steps = 8. To find the maximum sensing coverage rate, algorithm *Find-Max-Sen-Covrate* described in Section 4.5 is used. The value of *d_max_* is set to *Rc* and the value of *d_min_* is set to *Rc*/10. The value of *Max_SCL* is determined by the type of sensor nodes being deployed. For Type 1 sensor nodes, *Max_SCL* is updated with *Max_CCL* based on Equation (10) described in Section 4.3. For Type 2 sensor nodes with directional sensors, *Max_SCL* is set to 1 for *Rc/Rs* = 2 and 1.2. When *Rc/Rs* = 1/1.2, the value of *Max_SCL* is set to (*Rs/Rc*)^2^ = 1.44 to maintain the network connectivity.

After the deployment simulation is completed, we compare the simulation results of three coverage models in terms of the maximum sensing coverage rate and the usage of sensor nodes. The maximum sensing coverage rate is expressed as the actual sensing coverage rate by replacing the sensing areas of the deployed sensor nodes with the original sensing areas without modeling. The usage of sensor nodes is evaluated by the number of sensor nodes deployed at the maximum sensing coverage rate. In the following, we describe the simulation results in details based on the *Rc/Rs* ratios.

#### 5.1.1. Case 1: *Rc/Rs* = 2

Table 4 shows the maximum sensing coverage rates and the usage of sensor nodes for each type under three coverage models. From Table 4, we have the following remarks.

##### Remark 1

**Figure 11.**
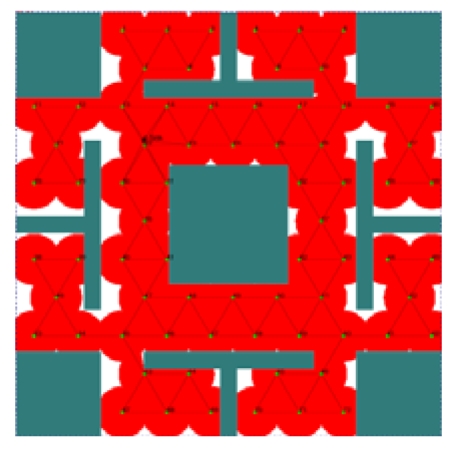
The sensing areas of Type 1 sensor nodes deployed with regular pattern in Case 1.

Regarding to the maximum sensing coverage rates, for Type 1 sensor nodes with round shapes of sensing areas, the proposed *Deploy-Random* algorithm can produce full sensing coverage rates for sensor nodes under the polygon or circular sector model while the disk model based deployment algorithm cannot. The reason is that the disk model based deployment algorithm uses a regular deployment pattern to achieve the maximum sensing coverage rate with the least number of sensor nodes. The regular deployment pattern cannot deal with the existence of boundaries and obstacles. Figure 11 shows the sensing area of Type 1 sensor nodes deployed with regular pattern. From Figure 11, we can see that some sensor nodes cannot be deployed over the obstacles or the positions around the boundaries. However, the maximum sensing coverage rate produced by the disk model based deployment algorithm is close to that of the proposed *Deploy-Random* algorithm.

**Figure 12.**
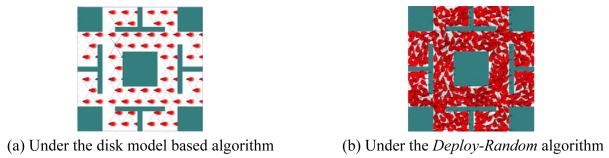
The sensing areas of Type 2 sensor nodes deployed by different algorithms in Case 1. (a) Under the disk model based algorithm (b) Under the *Deploy-Random* algorithm

For Type 2 sensor nodes, the maximum sensing coverage rate produced by the disk model based deployment algorithm is much lower than that of the proposed *Deploy-Random* algorithm. The reason is that the sensing area of a Type 2 sensor node is not a round shape (see Table 3); the sensing area represented by the disk model is much larger than the original one. In Figure 12, we show the sensing areas of WSNs produced by different deployment algorithms when Type 2 sensor nodes are deployed. From Figure 12, we can see that the disk model is not so accurate for sensor nodes with noncircular shapes of sensing areas. The difference of the maximum sensing coverage rates between the polygon model and the circular sector model is 2.14%/1.48% when the deployment area without/with obstacles. The reason is that the sensing area of a Type 2 sensor node under the circular sector model is larger than that under the proposed polygon model shown in Table 3. The sensing area under the proposed polygon model is more approximate to the actual one.

##### Remark 2

Regarding to the usage of sensor nodes, the disk model based deployment algorithm is less than that of the proposed *Deploy-Random* algorithm. For Type 1 sensor nodes with round shapes of sensing areas, the disk model based deployment algorithm can produce a very good deployment results in terms of the maximum sensing coverage rate and the usage of sensor nodes compared to the proposed *Deploy-Random* algorithm. The usage of sensor nodes between the circular sector model and the polygon model is very similar for Type 1 and Type 2 sensor nodes in this case.

#### 5.1.2. Case 2: *Rc/Rs* = 1.2

Table 5 shows the maximum sensing coverage rates and the usage of sensor nodes for each type under three coverage models. From Table 5, regarding to the maximum sensing coverage rates and the usage of sensor nodes, we have similar observations as those described in Remarks 1 and 2, respectively. The usage of sensor nodes under the circular sector model and the polygon model in this case is less than that in Case 1. The reason is the sensing area of the sensor node used in Case 2 is larger than that used in Case 1.

#### 5.1.3. Case 3: *Rc/Rs* = 1/1.2

Table 6 shows the maximum sensing coverage rates and the usage of sensor nodes for each type under these two deployment algorithms. From Table 6, regarding to the maximum sensing coverage rates and the usage of sensor nodes under these two algorithms, we have similar observations as those described in Remarks 1 and 2, respectively.

### 5.2. Simulation 2: the Polygon Model with Different Rotation Steps

In Simulation 2, we deploy Type 2 sensor nodes under the polygon model used in Simulation 1 by using the *Deploy-Random* algorithm with different rotation steps. Six rotation steps, 1, 4, 8, 16, 32, and 64, are simulated with three different *Rc/Rs* ratios, 2 (60/30), 1.2 (60/50), and 1/1.2 (60/72). The deployment configuration used in Simulation 2 is the same as that used in Simulation 1. Table 7 shows the maximum sensing coverage rates and the usage of sensor nodes by using algorithm *Find-Max-Sen-Covrate*. From Table 7, we have the following remark.

#### Remark 3

Regarding to the number of rotation steps used in the proposed *Deploy-Random* algorithm, in general, the maximum sensing coverage rate can be improved by increasing the number of rotation steps when the sensing areas of sensor nodes are noncircular. For example, for Case 1 without obstacles, the maximum coverage rate is always increased with the number of rotation steps from 1 to 64. For Case 2 and 3, the usage of the sensor nodes is reduced when the number of rotation steps is increased from 1 to 8 with/without obstacles. When the number of the rotation steps is more than 16, the differences of the maximum sensing coverage rate and the usage of the sensor nodes are not so significant.

### 5.3. Simulation 3: the Polygon Model with Different Number of Vertices

In Simulation 3, we used four sets of vertices to model the noncircular sensing area of a Type 2 sensor node used in Simulation 1. The number of vertices for each set is 16, 12, 9, and 4, respectively. The modeled areas are shown in Table 8. For Set 3, the vertices used to model the sensing area are the same as that of Type 2 used in Simulation 1. Three different *Rc/Rs* ratios, 2 (60/30), 1.2 (60/50), and 1/1.2 (60/72), are simulated. Table 8 shows the shape of the sensing area of the directional sensor for each set of sensor node under the polygon model. From Table 8, we can see different sensing areas of the directional sensor when different numbers of vertices are used under the polygon model. The deployment configuration used in Simulation 3 is the same as that used in Simulation 1. The number of rotation steps is fixed to 8.

Table 9 shows the maximum sensing coverage rates and the usage of sensor nodes of each set with 1,000 sensor nodes by using algorithm *Find-Max-Sen-Covrate*. From Table 9, we have the following remark.

#### Remark 4

If the number of sensor nodes is sufficient, that is, the maximum sensing coverage rate is over 99% and the usage of sensor nodes is less than 1,000, the difference of the maximum sensing coverage rates is very small among these four sets. For example, for Case 3, the maximum coverage rates for Set 1 ∼ 4 are all over 99.99%. However, the number of sensor nodes used for Set 4 (578/403 without/with obstacles) is larger than that used for Set 1 (392/272 without/with obstacles), Set 2 (386/290 without/with obstacles), and Set 3 (394/293 without/with obstacles). The reason is that the sensing area of the sensor node modeled by vertices in Set 4 is smaller than that of the sensor node modeled by vertices in Set 1 ∼ 3. When deploying sensor nodes in Set 4, two sensor nodes will be closer to each other compared to deploy sensor nodes in Set 1 ∼ 3 and more sensor nodes should be used if a deployment area is fully covered.

Table 10 shows the sensing coverage rates and the usage of sensor nodes of each set with 1000 sensor nodes by using algorithm *Find-Max-Sen-Covrate* under fixed values of *Max_CCL*. From Table 10, we have the following remarks.

#### Remark 5

If the value of *Max_CCL* is fixed and the number of sensor nodes is sufficient, we have similar observations as those of Remark 4. For example, for Case 2 and Case 3 with *Max_CCL* = 50.5, the difference of the sensing coverage rates is small for Set 1 ∼ 4. But the number of sensor nodes used for Set 4 is larger than that used for Set 1 ∼ 3.

#### Remark 6

If the value of *Max_CCL* is fixed and the number of sensor nodes is insufficient, that is, all deployable sensor nodes are used and the sensing coverage rate is less than 99%, the more vertices used to model a sensor node, the more sensing coverage rate can be achieved. For example, for Case 1 with *Max_CCL* = 50.5, the sensing coverage rates for Set 1 ∼ 4, without obstacles are 94.5%, 93.47%, 93.11%, and 82.97%, respectively. The reason is the same as that stated in Remark 4.

#### Remark 7

The value of *Max_CCL* affects the sensing coverage rate and the usage of sensor nodes for the same set of sensor node. If the number of sensor nodes is sufficient, a larger *Max_CCL* results in a larger sensor node usage. For example, for Case 2 with *Max_CCL* = 50.5 without/with obstacles, the number of sensor nodes used for Set 3 is 507/389 while that used with *Max_CCL* = 100 is 563/451. If the number of sensor node is insufficient, that is, all deployable sensor nodes are used, a larger *Max_CCL* results in a lower sensing coverage rate. For example, for Case 1 with *Max_CCL* = 50.5 without obstacles, the sensing coverage rate for Set 3 is 93.11% while that with *Max_CCL* = 100 is 83.53%.

## 6. Conclusions

In this paper, we have proposed a polygon model and the corresponding WSN deployment algorithm. The proposed polygon model can represent different shapes of the communication and sensing areas of sensor nodes. The four-step deployment algorithm utilizes the topology control mechanism and scoring process to determine the appropriate deployment positions in order to maintain the network connectivity and improve the gains of the sensing coverage rate. According to the simulation results, if the sensor node has noncircular sensing area, the maximum sensing coverage rate under the polygon model is higher than that of the disk model and the circular sector model. For the proposed WSN deployment algorithm, the maximum sensing coverage rate and the usage of sensor nodes are affected by the topology control parameters and the rotation steps of the sensor node. For the same type of sensor node modeled by a simple polygon (few vertices) and a complex polygon (many vertices), if the number of sensor nodes is sufficient, the difference of the maximum sensing coverage rates is very small. However, the usage of sensor nodes under the complex polygon is less than that under the simple polygon.

## Figures and Tables

**Table 1.** The definitions of symbols used in this paper.

*S_n_*	Sensor node *n*
*poly_C_*(*S_n_*)	The communication area of *S_n_* under the polygon model
(*Rc_i_*, *θc_i_*)	The *i*th vertex of *poly_C_*(*S_n_*)
*poly_S_*(*S_n_*)	The sensing area of *S_n_* under the polygon model
(*Rs_i_*, *θs_i_*)	The *i*th vertex of *poly_S_*(*S_n_*)
*Area*	A field
*Area_C_*(*S_n_*)	The communication area covered by *poly_C_*(*S_n_*) in a field
*Area_S_*(*S_n_*)	The sensing area covered by *poly_S_*(*S_n_*) in a field
*Loc*(*S_n_*)	The location of *S_n_* in a field
*Rot*(*S_n_*)	The rotation degree of *S_n_* counterclockwise from 0° in a field
*P_i_*	Point *i* of a field
*Rc*(*S_n_*, *P_i_*)	The communication range of *S_n_* in the direction of *P_i_*
*Rs*(*S_n_*, *P_i_*)	The sensing range of *S_n_* in the direction of *P_i_*
*θ*(*S_n_*, *P_i_*)	The angle between ray *S_n_P_i_* and the 0° ray originating at *S_n_*
*d*(*S_n_*, *P_i_*)	The Euclidean distance between *S_n_* and *P_i_*
Δ*P_a_P_b_P_c_*	The area formed by points *P_a_*, *P_b_*, and *P_c_*
*CCL*(*S_n_*, *P_i_*)	The communication coverage level of *S_n_* at *P_i_*
*SCL*(*S_n_*, *P_i_*)	The sensing coverage level of *S_n_* at *P_i_*
*W*	A wireless sensor network
*CR*(*Area,W*)	The sensing coverage rate of *W* on *Area*
*Square*(*P_i_*)	A square area centered at *P_i_*
*CR*(*Square*(*P_i_*), *W*)	The sensing coverage rate of *W* on *Square*(*P_i_*)
*NC*(*W*)	The network connectivity of *W*
*S_base_*	Base node
*Max*_*CCL*	The maximum threshold of the communication coverage level
*Max*_*SCL*	The maximum threshold of the sensing coverage level
*Score*(*P_i_*)	The score of a candidate position *P_i_*
*Rot_step_*	The rotation steps of *S_n_*
*Rc*	The maximum communication range of a sensor node
*Rs*	The maximum sensing range of a sensor node
*d_max_*	The maximum distance between sensor nodes

**Table 2.** The coverage areas of omnidirectional antenna/sensor under different models.

**Coverage area**	**Disk/Circular sector model**	**Polygon model**
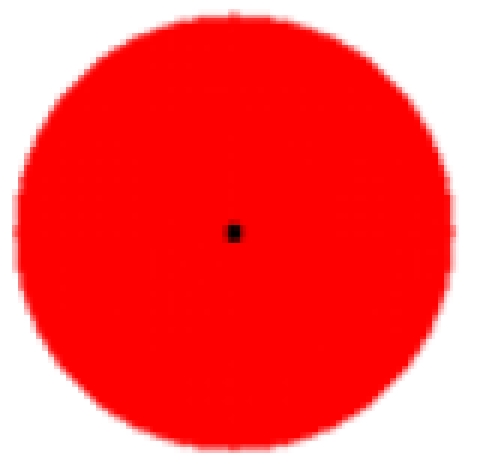 **antenna** *Rc* = 60	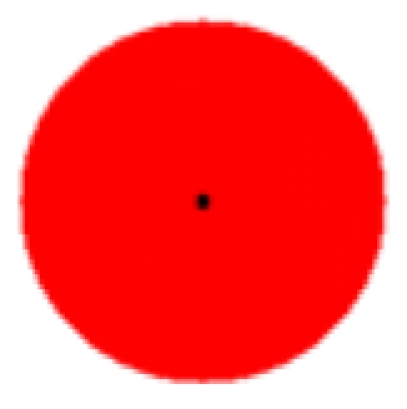 central angle= 360° radius = 60	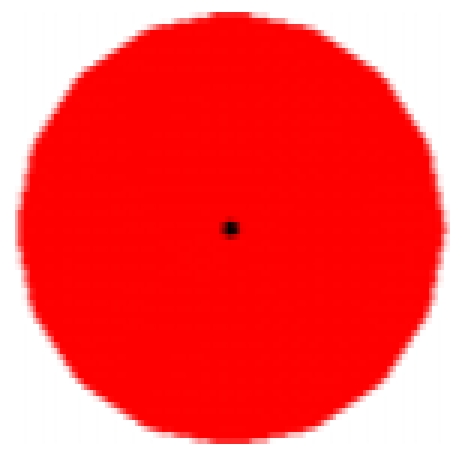 [(60, 0°), (60, 22.5°), (60, 45°), (60, 67.5°), (60, 90°), (60, 112.5°), (60, 135°), (60, 157.5°), (60, 180°), (60, 202.5°), (60, 225°), (60, 247.5°), (60, 270°), (60, 292.5°), (60, 315°), (60, 337.5°)]
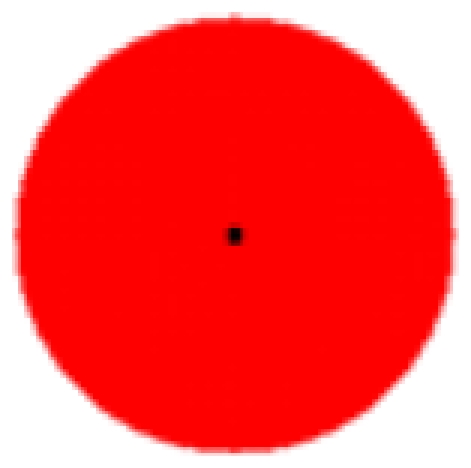 **sensor** *Rs* = 30 (Case 1: *Rc/Rs* = 2) 50 (Case 2: *Rc/Rs* = 1.2) 72 (Case 3: *Rc/Rs* = 1/1.2)	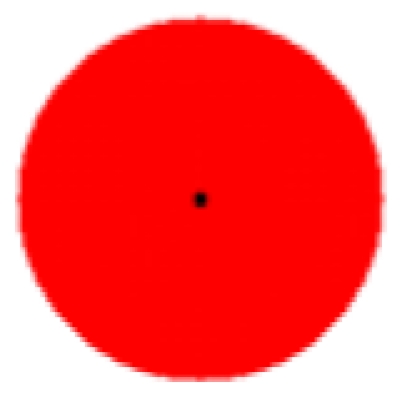 angle= 360° radius = 30 (Case 1)50 (Case 2), 72 (Case 3)	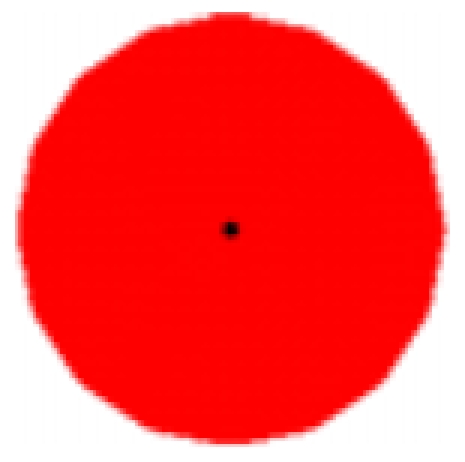 [(*Rs*_1_, 0°), (*Rs*_2_, 22.5°), (*Rs*_3_, 45°), (*Rs*_4_, 67.5°), (*Rs*_5_, 90°), (*Rs*_6_, 112.5°), (*Rs*_7_, 135°), (*Rs*_8_, 157.5°), (*Rs*_9_, 180°), (*Rs*_10_, 202.5°), (*Rs*_11_, 225°), (*Rs*_12_, 247.5°), (*Rs*_13_, 270°), (*Rs*_14_, 292.5°), (*Rs*_15_, 315°), (*Rs*_16_, 337.5°)] *Rs_i_* = 30 (Case 1), 50 (Case 2), 72 (Case 3)

**Table 3.** The coverage areas of directional sensor under different models.

**Coverage area**	**Disk model**	**Circular sector model**	**Polygon model**
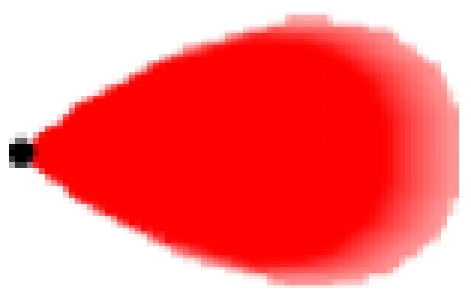	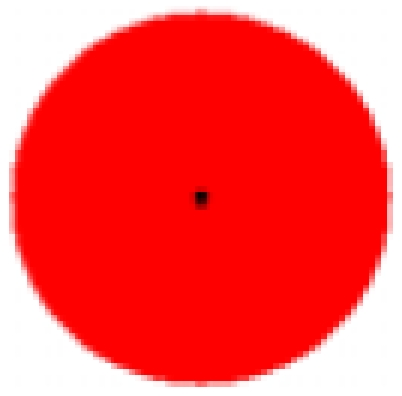	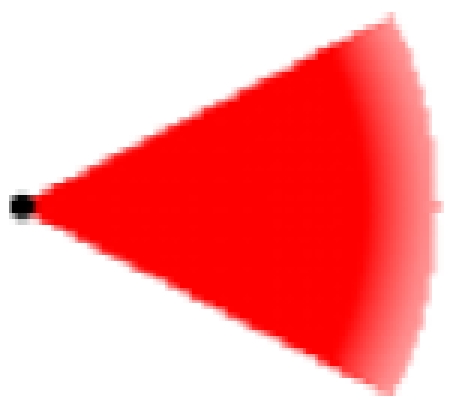	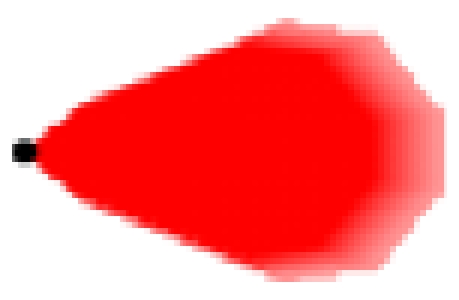
*Rs* = 30 (Case 1: *Rc/Rs* = 2) 50 (Case 2: *Rc/Rs* = 1.2) 72 (Case 3: *Rc/Rs* = 1/1.2)	central angle = 360° radius = 30 (Case 1) 50 (Case 2) 72 (Case 3)	central angle = 53.2° radius = 30 (Case 1) 50 (Case 2) 72 (Case 3)	[(30, 4.9°), (26.5, 18.3°), (20.7, 26.6°), (6, 38.7°), (0, 180°),(6, 321.3°), (20.7, 333.4°), (26.5, 341.7°), (30, 355.1°)] (Case 1) [(50, 4.9°), (44.1, 18.3°), (34.6, 26.6°), (10, 38.7°), (0, 180°),(10, 321.3°), (34.6 333.4°), (44.1, 341.7°), (50, 355.1°)] (Case 2) [(72, 4.9°), (63.6, 18.3°), (49.68, 26.6°), (14.4, 38.7°), (0, 180°), (14.4, 321.3°), (49.68, 333.4°), (63.6, 341.7°), (72, 355.1°)] (Case 3)

**Table 4.** The maximum sensing coverage rates and the usage of sensor nodes for each type in Case 1.

	**Disk model**		**Circular sector model** **(rotation steps: 8)**		**Polygon model** **(rotation steps: 8)**	
**Deployment area without obstacles**	**coverage**	**#sensor**	**coverage**	**#sensor**	**coverage**	**#sensor**

Type 1	0.9752	105	1 (*Max_CCL* = 4.09375)	224	1 (*Max_CCL* = 4.09375)	229
Type 2	0.1556	105	0.9298 (*Max_CCL* = 48.1797)	992	0.9512 (*Max_CCL* = 40.832)	1000

**Deployment area with obstacles**	**coverage**	**#sensor**	**coverage**	**#sensor**	**coverage**	**#sensor**

Type 1	0.9073	72	1 (*Max_CCL* = 4.09375)	173	1 (*Max_CCL* = 4.09375)	179
Type 2	0.1411	72	0.9706 (*Max_CCL* = 100)	920	0.9854 (*Max_CCL* = 100)	930

**Table 5.** The maximum sensing coverage rates and the usage of sensor nodes for each type in Case 2.

	**Disk model**		**Circular sector model** **(rotation steps: 8)**		**Polygon model** **(rotation steps: 8)**	
**Deployment area without obstacles**	**coverage**	**#sensor**	**coverage**	**#sensor**	**coverage**	**#sensor**

Type 1	0.9898	72	1 (*Max_CCL* = 1.38672)	85	1 (*Max_CCL* = 1.38672)	84
Type 2	0.2948	72	0.9792(*Max_CCL* =100)	573	0.9943(*Max_CCL* =100)	563

**Deployment area with obstacles**	**coverage**	**#sensor**	**coverage**	**#sensor**	**coverage**	**#sensor**

Type 1	0.9732	48	1 (*Max_CCL* = 1.38672)	72	1 (*Max_CCL* = 1.38672)	72
Type 2	0.2144	48	0.9874(*Max_CCL* = 100)	444	0.9954(*Max_CCL* = 100)	451

**Table 6.** The maximum sensing coverage rates and the usage of sensor nodes for each type in Case 3.

	**Disk model**		**Circular sector model** **(rotation steps: 8)**		**Polygon model** **(rotation steps: 8)**	
**Deployment area without obstacles**	**coverage**	**#sensor**	**coverage**	**#sensor**	**coverage**	**#sensor**

Type 1	1	68	1 (*Max_CCL* = 1.38672)	74	1 (*Max_CCL* = 1.38672)	69
Type 2	0.5434	68	0.9946 (*Max_CCL* = 100)	391	1 (*Max_CCL* = 100)	394

**Deployment area with obstacles**	**coverage**	**#sensor**	**coverage**	**#sensor**	**coverage**	**#sensor**

Type 1	0.9748	45	1 (*Max_CCL* = 1.38672)	55	1 (*Max_CCL* = 1.38672)	54
Type 2	0.3953	45	0.9952 (*Max_CCL* = 100)	305	0.9999 (*Max_CCL* =100)	293

**Table 7.** The maximum sensing coverage rates and the usage of sensor nodes in Simulation 2.

**Deployment area**	**Rotation steps: 1**		**Rotation steps: 4**		**Rotation steps: 8**	
**without obstacles**	**coverage**	**#sensor**	**coverage**	**#sensor**	**coverage**	**#sensor**

Case 1: *Rc/Rs* = 2	0.9258 (*Max_CCL* =24.2031)	992	0.9344 (*Max_CCL* =41.2188)	997	0.9512 (*Max_CCL* = 40.832)	1000
Case 2: *Rc/Rs* = 1.2	0.9634 (*Max_CCL* =35.0312)	993	0.9880 (*Max_CCL* =100)	720	0.9943 (*Max_CCL* =100)	563
Case 3: *Rc/Rs* = 1/1.2	0.988209 (*Max_CCL* =42.3789)	1000	1 (*Max_CCL* =95.3594)	524	1 (*Max_CCL* = 100)	394

**with obstacles**	**coverage**	**#sensor**	**coverage**	**#sensor**	**coverage**	**#sensor**

Case 1: *Rc/Rs* = 2	0.923019 (*Max_CCL* =50.8867)	988	0.969716 (*Max_CCL* =100)	963	0.9854 (*Max_CCL* = 100)	930
Case 2: *Rc/Rs* = 1.2	0.95657 (*Max_CCL* =96.9062)	999	0.987844 (*Max_CCL* =100)	536	0.9954 (*Max_CCL* =100)	451
Case 3: *Rc/Rs* = 1/1.2	0.97462 (*Max_CCL* =100)	801	0.999915 (*Max_CCL* =100)	387	0.9999 (*Max_CCL* =100)	293

	**Rotation steps: 16**	**Rotation steps: 32**	**Rotation steps: 64**

**without obstacles**	**coverage**	**#sensor**	**coverage**	**#sensor**	**coverage**	**#sensor**

Case 1: *Rc/Rs* = 2	0.958755 (*Max_CCL* = 41.9922)	990	0.960028 (*Max_CCL* = 41.6055)	1000	0.965105 (*Max_CCL* =38.8984)	1000
Case 2: *Rc/Rs* = 1.2	0.9959 (*Max_CCL* = 100)	537	0.9960 (*Max_CCL* = 100)	536	0.9971 (*Max_CCL* = 100)	537
Case 3: *Rc/Rs* = 1/1.2	1 (*Max_CCL* =85.6914)	369	0.999996 (*Max_CCL* =100)	396	0.999992 (*Max_CCL* =100)	390

**with obstacles**	**coverage**	**#sensor**	**coverage**	**#sensor**	**coverage**	**#sensor**

Case 1: *Rc/Rs* = 2	0.991359 (*Max_CCL* = 100)	921	0.990761 (*Max_CCL* = 100)	912	0.991908 (*Max_CCL* = 100)	926
Case 2: *Rc/Rs* = 1.2	0.997216 (*Max_CCL* = 100)	440	0.997367 (*Max_CCL* = 100)	415	0.996999 (*Max_CCL* = 100)	414
Case 3: *Rc/Rs* = 1/1.2	0.999994 (*Max_CCL* =100)	306	0.999982 (*Max_CCL* =100)	291	1 (*Max_CCL* =100)	292

**Table 8.** The configuration of Type 2 sensor node in Simulation 3.

**Coverage area**	**Polygon model** **(Set 1: 16 vertices)**	**Polygon model** **(Set 2: 12 vertices)**	**Polygon model** **(Set 3: 9 vertices)**	**Polygon model** **(Set 4: 4 vertices)**
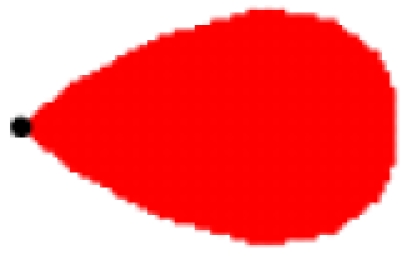	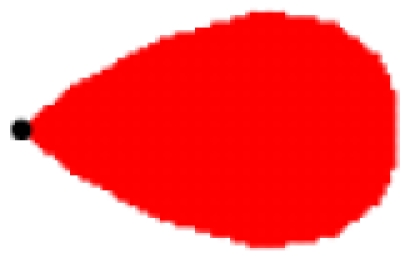	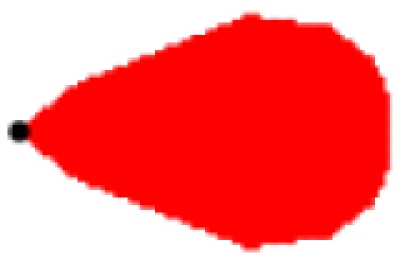	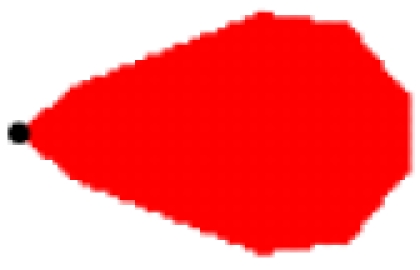	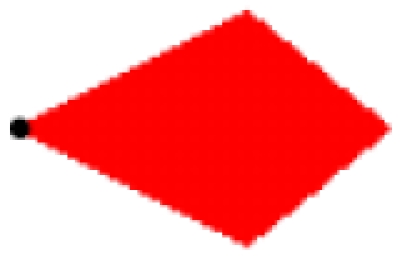
*Rs* = 30 (Case 1: *Rc/Rs* = 2) 50 (Case 2: *Rc/Rs* = 1.2) 72 (Case 3: *Rc/Rs* = 1/1.2)	[(29.9, 0°), (30, 4.9°), (29, 11.7°), (26.5, 18.3°), (24.3, 22°), (20.7, 26.6°), (13.5, 32.4°), (6, 38.7°), (0, 180°), (6, 321.3°), (13.5, 327.6°), (20.7, 333.4°), (24.3, 338°), (26.5, 341.7°), (29, 348.3°), (30, 30.1°)] (Case 1) [(49.9, 0°), (50, 4.9°), (48.3, 11.7°), (44.1, 18.3°), (40.6, 22°), (34.6, 26.6°), (22.3, 32.4°), (10, 38.7°), (0, 180°), (10, 321.3°), (22.3, 327.6°), (34.6, 333.4°), (40.6, 338°), (44.1, 341.7°), (48.3, 348.3°), (50, 355.1°)] (Case 2) [(72, 0°), (72, 4.9°), (69.6, 11.7°), (63.6, 18.3°), (58.32, 22°), (49.68, 26.6°), (32.4, 32.4°), (14.4, 38.7°), (0, 180°), (14.4, 321.3°), (32.4, 327.6°), (49.68, 333.4°), (58.32, 338°), (63.6, 341.7°), (69.6, 348.3°), (72, 355.1°)] (Case 3)	[(29.9, 0°), (30, 4.9°), (29, 11.7°), (26.5, 18.3°),(20.7, 26.6°), (6, 38.7°), (0, 180°), (6, 321.3°), (20.7, 333.4°), (26.5, 341.7°), (29, 348.3°), (30.1°)] (Case 1) [(49.9, 0°), (50, 4.9°), (48.3, 11.7°), (44.1, 18.3°), (34.6, 26.6°), (10, 38.7°), (0, 180°), (10, 321.3°), (34.6, 333.4°), (44.1, 341.7°), (48.3, 348.3°), (50, 355.1°)] (Case 2) [(71.76, 0°), (72, 4.9°), (69.6, 11.7°), (63.6, 18.3°), (49.68, 26.6°), (14.4, 38.7°), (0, 180°), (14.4, 321.3°), (49.68, 333.4°), (63.6, 341.7°), (69.6, 348.3°), (72, 355.1°)] (Case 3)	[(30, 4.9°), (26.5, 18.3°), (20.7, 26.6°), (6, 38.7°), (0, 180°),(6, 321.3°), (20.7, 333.4°), (26.5, 341.7°), (30, 355.1°)] (Case 1) [(50, 4.9°), (44.1, 18.3°), (34.6, 26.6°), (10, 38.7°), (0, 180°),(10, 321.3°), (34.6 333.4°), (44.1, 341.7°), (50, 355.1°)] (Case 2) [(72, 4.9°), (63.6, 18.3°), (49.68, 26.6°), (14.4, 38.7°), (0, 180°),(14.4, 321.3°), (49.68, 333.4°), (63.6, 341.7°), (72, 355.1°)] (Case 3)	[(30, 0°), (20.7, 26.6°), (0, 180°), (20.7, 333.4°)](Case 1)[(50, 0°), (34.6, 26.6°), (0, 180°), (34.6 333.4°)](Case 2)[(72, 0°), (49.68, 26.6°), (0, 180°), (49.68, 333.4°)](Case 3)

**Table 9.** The maximum sensing coverage rates and the usage of sensor nodes in Simulation 3.

	**Polygon model** **(Set 1: 16 vertices)** **(rotation steps: 8)**		**Polygon model** **(Set 2: 12 vertices)** **(rotation steps: 8)**		**Polygon model** **(Set 3: 9 vertices)** **(rotation steps: 8)**		**Polygon model** **(Set 4: 4 vertices)** **(rotation steps: 8)**	
**Deployment area without obstacles**	**coverage**	**#sensor**	**coverage**	**#sensor**	**coverage**	**#sensor**	**coverage**	**#sensor**

Case 1: *Rc/Rs* = 2	0.95(*Max_CCL* = 45.8594)	1000	0.9528(*Max_CCL* = 41.9922)	1000	0.9512(*Max_CCL* = 40.832)	1000	0.9518(*Max_CCL* = 26.1367)	1000
Case 2: *Rc/Rs* = 1.2	0.9911(*Max_CCL* = 100)	519	0.9927(*Max_CCL* = 100)	548	0.9943(*Max_CCL* =100)	563	0.9996(*Max_CCL* =100)	733
Case 3: *Rc/Rs* = 1/1.2	1 (*Max_CCL* = 98.0664)	392	1 (*Max_CCL* = 100)	386	1 (*Max_CCL* = 100)	394	1 (*Max_CCL* = 100)	578

**Deployment area with obstacles**	**coverage**	**#sensor**	**coverage**	**#sensor**	**coverage**	**#sensor**	**coverage**	**#sensor**

Case 1: *Rc/Rs* = 2	0.9789(*Max_CCL* = 100)	880	0.9861(*Max_CCL* = 100)	928	0.9854(*Max_CCL* = 100)	930	0.9916(*Max_CCL* = 55.5273)	1000
Case 2: *Rc/Rs* = 1.2	0.9926(*Max_CCL* = 100)	414	0.9952(*Max_CCL* = 100)	454	0.9954(*Max_CCL* =100)	451	0.9995(*Max_CCL* =100)	573
Case 3: *Rc/Rs* = 1/1.2	1 (*Max_CCL* = 86.4648)	272	0.9999(*Max_CCL* = 100)	290	0.9999(*Max_CCL* =100)	293	0.9999(*Max_CCL* =100)	403

**Table 10.** The sensing coverage rates and the usage of sensor nodes under fixed values of *Max_CCL* in Simulation 3.

	**Polygon model** **(16 vertices)** **(rotation steps: 8)**		**Polygon model** **(12 vertices)** **(rotation steps: 8)**		**Polygon model** **(9 vertices)** **(rotation steps: 8)**		**Polygon model** **(4 vertices)** **(rotation steps: 8)**	
	*Max_CCL* = 50.5							

**Deployment area without obstacles**	**coverage**	**#sensor**	**coverage**	**#sensor**	**coverage**	**#sensor**	**coverage**	**#sensor**

Case 1: *Rc/Rs* = 2	0.945	1000	0.9347	1000	0.9311	1000	0.8297	1000
Case 2: *Rc/Rs* = 1.2	0.9797	470	0.9865	485	0.9912	507	0.9981	634
Case 3: *Rc/Rs* = 1/1.2	0.9999	343	1	354	1	371	1	490

**Deployment area with obstacles**	**coverage**	**#sensor**	**coverage**	**#sensor**	**coverage**	**#sensor**	**coverage**	**#sensor**

Case 1: *Rc/Rs* = 2	0.9605	757	0.9662	792	0.9676	802	0.9895	973
Case 2: *Rc/Rs* = 1.2	0.9851	378	0.9886	383	0.9913	389	0.9980	496
Case 3: *Rc/Rs* = 1/1.2	0.9999	282	0.9999	288	0.9998	285	1	372

	*Max_CCL* = 100							

**Deployment area without obstacles**	**coverage**	**#sensor**	**coverage**	**#sensor**	**coverage**	**#sensor**	**coverage**	**#sensor**

Case 1: *Rc/Rs* = 2	0.862	1000	0.8476	1000	0.8353	1000	0.7186	1000
Case 2: *Rc/Rs* = 1.2	0.9911	519	0.9927	548	0.9943	563	0.9996	733
Case 3: *Rc/Rs* = 1/1.2	1	388	1	386	1	394	1	578

**Deployment area with obstacles**	**coverage**	**#sensor**	**coverage**	**#sensor**	**coverage**	**#sensor**	**coverage**	**#sensor**

Case 1: *Rc/Rs* = 2	0.9789	880	0.9861	928	0.9854	930	0.8651	1000
Case 2: *Rc/Rs* = 1.2	0.9926	414	0.9952	454	0.9954	451	0.9995	573
Case 3: *Rc/Rs* = 1/1.2	1	293	0.9999	290	0.9999	293	0.9999	403
